# Home-Based Care for Hypertension in Rural South Africa

**DOI:** 10.1056/NEJMoa2509958

**Published:** 2025-09-01

**Authors:** Mark J. Siedner, Nombulelo Magula, Lusanda Mazibuko, Nsika Sithole, Alison Castle, Siyabonga Nxumalo, Thabang Manyaapelo, Shafika Abrahams-Gessel, Dickman Gareta, Joanna Orne-Gliemann, Kathy Baisley, Max Bachmann, Thomas A. Gaziano

**Affiliations:** 1 Africa Health Research Institute, KwaZulu-Natal, South Africa; 2 Division of Internal Medicine, University of KwaZulu-Natal, Durban, KwaZulu-Natal, South Africa; 3 Division of Infectious Diseases and Medical Practice Evaluation Center, Department of Medicine, Massachusetts General Hospital, Boston, Massachusetts, United States of America; 4 Harvard Medical School, Boston, Massachusetts, United States of America; 5 Harvard TH Chan School of Public Health, Boston, Massachusetts, United States of America; 6 Institute of Social and Preventive Medicine, University of Bern, Bern, Switzerland; 7 School of Laboratory Medicine and Medical Sciences, University of KwaZulu Natal, Durban, South Africa; 8 University of Bordeaux, Bordeaux Population Health Centre, Bordeaux, France; 9 Faculty of Epidemiology and Population Health, London School of Hygiene & Tropical Medicine, London, United Kingdom; 10 Norwich Medical School, University of East Anglia, Norwich, United Kingdom; 11 Division of Cardiology, Brigham and Women’s Hospital, Boston, Massachusetts, United States of America

## Abstract

**Background:**

Hypertension control rates remain poor worldwide, particularly in low-resource settings.

**Methods:**

We conducted an open-label, individually-randomized controlled trial of home-based hypertension care in South Africa. Adults over 18 with hypertension were randomized to: clinic-based management (SOC arm); home-based blood pressure (BP) self-monitoring, community health worker visitation for data collection and medication delivery, and remote, nurse-led care, assisted by a mobile application with decision support (CHW arm), and an enhanced CHW arm, whereby BP machines transmitted readings automatically (eCHW+ arm). The primary outcome was systolic BP at 6 months. Secondary outcomes were systolic BP at 12 months and hypertension control at 6 and 12 months. Safety endpoints included hospitalizations, deaths, and retention in care.

**Results:**

We randomized 774 individuals. The mean age was 62, 76% were women, 14% had diabetes and 47% had HIV. Compared to SOC, mean systolic BP at 6 months was lower in the CHW arm (−7.9mm Hg, 95%CI −10.5, −5.3, P<0.001) and eCHW+ arm (−9.1mmHg, 95%CI −11.7, −6.4, P<0.001). In the SOC arm, hypertension control at 6 months was 57.6%, compared to 76.9% in the CHW arm (RR 1.33 vs SOC, 95%CI 1.18–1.51), and 82.8% in the eCHW+ arm (RR 1.44 vs SOC, 95%CI 1.28–1.62). Improved BP with home-based care appeared to persist at 12 months. Severe adverse events (2.7%) and deaths (1.0%) were uncommon and similar across arms. Retention in care remained >95% in both intervention arms.

**Conclusions:**

Home-based hypertension care led to significant reductions in systolic BP and improvements in hypertension control in South Africa.

## Background

Elevated blood pressure (BP) is the leading risk factor for preventable mortality, responsible for approximately 10 million deaths each year.^1^ Although numerous low cost, effective therapies are available, disease control rates are poor, particularly among populations with structural barriers to healthcare.^2–4^ In the public sector of South Africa, lack of patient self-efficacy, over-crowded clinics, inconsistent availability of sphygmomanometers, and the costs of transportation to clinic and missed work are commonly cited contributors to sub-optimal outcomes.^5,6^ Home-based BP management with remote monitoring has been proposed to address these barriers,^7^ but data on the efficacy of such programs are scarce.

## Methods

### Patient and Public Involvement in Intervention Development

The intervention tested in this trial was developed in partnership with people living with hypertension and the Department of Health in South Africa. Full details of the formative work that motivated intervention development and adaptation have been published previously.^6^ In brief, input from partners resulted in three major design elements: 1) direct provision of BP machines to patients to promote self-efficacy, 2) remote disease monitoring to reduce patient costs, decongest clinics and support nurses with decision making, and 3) the selection of community health workers (CHWs) to facilitate care to align with National Department of Health priorities.^8^

### Trial Design and Eligibility Criteria

We conducted a three-arm, parallel group, open label randomized controlled trial. The trial was designed and implemented as a superiority trial with two intervention arms individually compared to a standard of care arm. Study protocol and analysis plan versions and changes are available in the Supplemental Materials.

Detailed methods have been published previously.^9^ We recruited individuals from two public-sector, primary care clinics in rural KwaZulu-Natal. Individuals were screened with BP measurements by study nurses located at the clinics and eligible if they were aged 18 or over and had evidence of uncontrolled hypertension as defined by the South African Department of Health Guidelines, which requires two BP measurements >140 mmHg systolic and/or >90 mmHg diastolic, taken a minimum of 6 months apart, with diet and lifestyle advice given in the interim.^10^ Provision of lifestyle advice was not recorded in patient files, so was not used as an eligibility criterion for this study. Eligibility criteria also included residence in the catchment area of study clinics, to enable CHW visitation for home-based monitoring, and plans to remain in the area for at least 24 months. We excluded those who required immediate referral to a physician, according to South African Department of Health guidelines, including pregnancy or breast feeding; severely high BP (>180mmHg systolic or 100mmHg diastolic) accompanied by symptoms; reduced renal function, as determined by an estimated glomerular filtrate rate (GFR) <60ml/minute/1.73 m^2^ calculated using the CKD-EPI equation from a point-of-care creatinine test on the day of enrollment;^11^ or current use of three or more anti-hypertensives at maximal dose. Those eligible who provided informed consent were randomized 1:1:1 in blocks of nine to one of the three trial arms using the randomization module in REDCap.^9,12^ Randomization was stratified by clinic and current use of hypertension treatment at enrollment. The study statistician generated the randomization table. Only the data manager had access to the locked randomization table. After randomization, both participants and clinic staff were aware of treatment allocation.

### Study Arms

Randomized participants in all three arms were seen by a nurse on the day of enrollment to determine initial hypertension therapy. Nurses involved in the program received training prior to study start on best practices for hypertension care according to South African Department of Health hypertension guidelines.^13^ The three principle anti-hypertensive therapies available in the public sector in South Africa and used in this study were hydrochlorothiazide, lisinopril, and amlodipine. All therapies are provided by the Department of Health free of charge to participants. In the standard of care (SOC) arm, participants were asked to return to clinic approximately monthly for BP measurements, titration of antihypertensive therapy according to the national guidelines, and collection of medications from the clinic pharmacies.

In the CHW arm, participants received an automated BP machine (Omron Digital M3. Kyoto, Japan), were trained on its use by CHWs, and advised to measure their BP daily. Individuals with an arm circumference ≥42cm received a large-sized cuff. CHWs visited participants within one week of enrollment and approximately monthly thereafter to record BP readings into a mobile health application on their phones (iMarketing Consultants, Windhoek, Namibia, see Supplementary materials for full details and screenshots of the application). Clinic nurses in the CHW arms received monthly prompts from the application to review participant data and enter prescribing information. The application was programmed with the treatment algorithm from the national treatment guidelines and made recommendations based on the mean blood pressure readings from the past two weeks. Once the nurse entered a decision into the application, it produced an electronic prompt for a clinic staff member to fill the prescription. Once the prescription was filled, CHWs received a prompt via the application on their phone to retrieve the medications and deliver them to participants’ homes.

In the second intervention arm (eCHW+), participants received a BP machine with short message service text message capability (BlipCare, Carematrix Inc, Chicago, IL). These machines transmitted BP data directly to the application used by nurses for BP clinical decision-making. Participants in the eCHW+ arm were visited by CHWs to ensure the BP machines were functional and to deliver medicines prescribed by nurses.

### Data Collection and Study Outcomes

At enrollment, we collected data on socio-demographics and medical history.^14^ Study staff not involved with the intervention program and blinded to study arm conducted home visits at 6 and 12 months after enrollment in all three arms to collect outcome data. At these visits, study staff conducted three BP measurements, 5 minutes apart, with participants in a seated position using automated sphygmomanometers. BP readings were taken as the average of the second and third readings. At follow-up visits, we also collected data on adverse events and hospitalizations in the past six months.

### Sample Size

Based on a recent population-based study of BP in the area, we anticipated mean BP at baseline would be 150/95 mmHg, with a standard deviation (SD) of 19 mmHg.^15^ With a target enrollment of 774 participants (258 per arm), we had >80% power to detect a 5 mmHg difference between arms in mean systolic BP at 6 months, allowing for 20% loss to follow-up, a correlation between baseline and follow-up measurements of 0.5, and a two-tailed alpha of 2.5% to account for multiple comparisons between the SOC and both intervention arms.

### Statistical Analyses

We used an intention-to-treat approach for all analyses. In our primary analysis, those with missing data were censored. Our primary outcome of interest was the difference between arms in systolic BP at 6 months. The secondary outcome of interest was the difference between arms in the proportion of participants with hypertension control at 6 months, defined by a systolic BP <140mm Hg and a diastolic BP <90mm Hg. Safety outcomes included adverse events and retention in hypertension care, defined as an interaction with a healthcare worker (i.e. nurse, physician or CHW) for hypertension care within the past 3 months. Additional pre-specified secondary outcomes were the difference between arms in systolic BP at 12 months and proportion with hypertension control at 12 months.

For the primary outcome, we fit linear regression models to estimate the difference between arms in mean systolic BP at 6 months. The model included terms for treatment arm, baseline systolic BP and randomization strata (clinic and use of hypertension medication at enrollment). We used the same approach for the analysis at the 12-month timepoint. We calculated the proportion of participants with hypertension control at 6 and 12 months in each treatment arm, and fit separate logistic regression models at each timepoint to estimate the odds ratio (OR) and derive the relative risk (RR) using marginal standardization for the effect of each intervention on hypertension control, compared with SOC.^16^

In exploratory analyses, we estimated the main treatment effect at 6 months in pre-specified sub-groups of interest by fitting an interaction term between treatment arm and the covariate (e.g. sex, age <60 versus ≥60, systolic BP at enrollment 140–160mm Hg versus ≥160mm Hg, and HIV status). In post-hoc analyses, we also considered effect modification by body mass index (≥30 vs <30), renal function (GFR >72 vs <72, the cohort median), sociodemographic variables, including employment status and whether participants had running water in their homes. Additional sensitivity analyses are described in the Supplementary Materials. Multiplicity control was only applied to the primary outcome, using the Bonferroni adjustment by dividing the pre-specified alpha of 5% by two to compare both intervention arms versus the SOC. For all secondary and exploratory outcomes, point estimates with 95% CIs are reported only, and these estimates were not controlled for multiplicity. Therefore, interpretation of the remaining analyses should be made with caution.

Finally, to compare safety outcomes, we summarized the number of total and severe adverse events by study arm. We then calculated the proportion of participants retained in care at 6 and 12 months. To do so, we conducted chart reviews for all study participants and defined retention in care as participants who had either 1) record of a clinic visit or CHW home visit in the 3 months preceding the timepoint or 2) an active prescription for anti-hypertension therapy in their medical chart at the timepoint.

### Ethical Considerations

The study was designed by the investigators with input from local Department of Health colleagues and the Africa Health Research Institute community advisory board. The investigators gathered and analyzed the data, vouch for the analysis, authored the manuscript, and made the decision to publish the manuscript. The trial was approved by the University of KwaZulu-Natal Biomedical Research Ethics Committee, the Institutional Review Board of Mass General Brigham and by the South African Health Products Regulatory Authority. All participants provided signed informed consent. A data safety monitoring board conducted an interim assessment after 50% of study participants had completed 6 months of participation in the trial. The committee recommended continuation of the trial based on review of this data.

## Results

Between November 2022 and June 2024, we screened 910 individuals with elevated BP and a record of ≥1 prior elevated reading 6 months prior. Of these, 136 did not meet inclusion criteria (Figure S1). The remaining 774 individuals consented for participation and were randomized to the standard of care (n=259), CWH (n=257), or eCHW+ (n=258) arms, respectively. Of these 762 (98%) completed the 6-month follow-up visit and were included in the primary intention-to-treat analysis. All individuals in the two intervention arms and no individuals in the standard of care arm were assigned a CHW for home visitation and received a BP cuff.

Participant characteristics are summarized in Table 1. One hundred percent of participants were of black African descent, reflecting the demographics of people with hypertension in this rural region South Africa (Table S1). At enrollment, the mean age was 62 years (SD 12) and 76% (588/774) were women. The mean systolic BP was 147mm Hg (SD 17) and approximately 20% (156/774) had a systolic BP >160mmHg. Forty-seven percent of participants (360/774) were living with HIV; whereas 14% (105/774) had diabetes and 45% (351/774) had a body mass index >30 kilogram/meter^2^. Eleven percent (87/774) were employed and 15% (112/774) had access to running water in their household. Sociodemographic and clinical characteristics were similar between arms.

**Figure 1.**
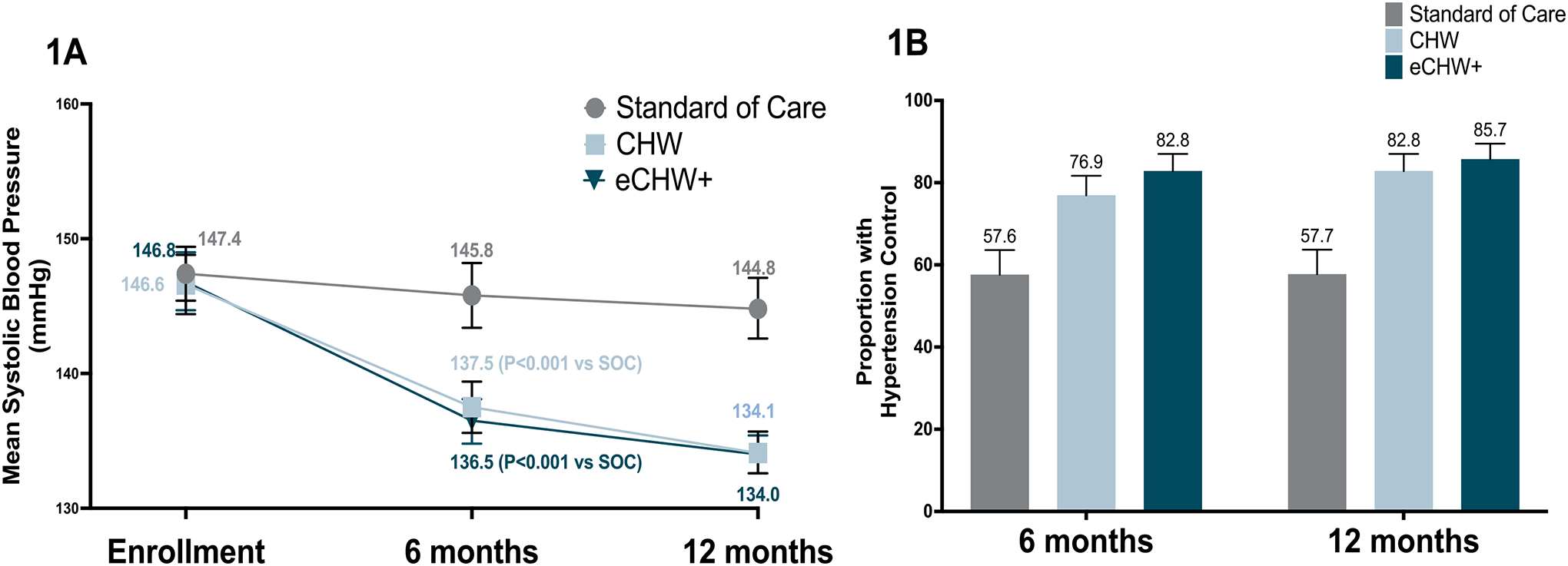
Mean systolic blood pressure (95%CI) (1A) and proportion with hypertension control (95%CI) (1B) in standard of care, community health worker (CHW) and enhanced community health worker (eCHW+) arms at 6 and 12 months

Mean systolic BP was similar at baseline and 6 months in the SOC arm (−1.9 mmHg, 95%CI −4.2, 0.4, Table 2, Figure 1A). By contrast, there was a reduction in mean systolic BP in the CHW arm (−9.1mmHg, 95%CI −11,1, −6.8, difference vs SOC −7.9mmHg, 95%CI −10.5, −5.3, P-value<0.001) and in the eCHW+ arm (−10.5mmHg, 95%CI −12.8, −8.2, difference vs SOC −9.1mmHg, 95%CI −11.7, −6.4, P-value<0.001). The proportion of participants achieving hypertension control based on South African Department of Health definitions at 6 months was 57.6% in the SOC arm (95%CI 51.5, 63.6), 76.8% in the CHW arm (95%CI 71.2, 81.7), and 82.8% in the eCHW+ arm (95%CI 77.7, 87.0, Figure 1B). The reduction in mean systolic BP in both intervention arms appeared to be sustained at 12 months (Table 2, Figure 1A). Similar patterns of diastolic blood pressure reductions were observed (Table S4) and results were similar in sensitivity analyses in which we adjusted for confounders and used the last BP reading carried forward for those with missing BP data (Tables S2 and S3).

There were 20 severe adverse events in 21 participants, including 8 deaths and 13 hospitalizations (Table 2, Table S5). Events were similar across arms and none were deemed related to study procedures. Retention in care remained >95% in both intervention arms at 6 and 12 months (Table 2).

**Figure 2.**
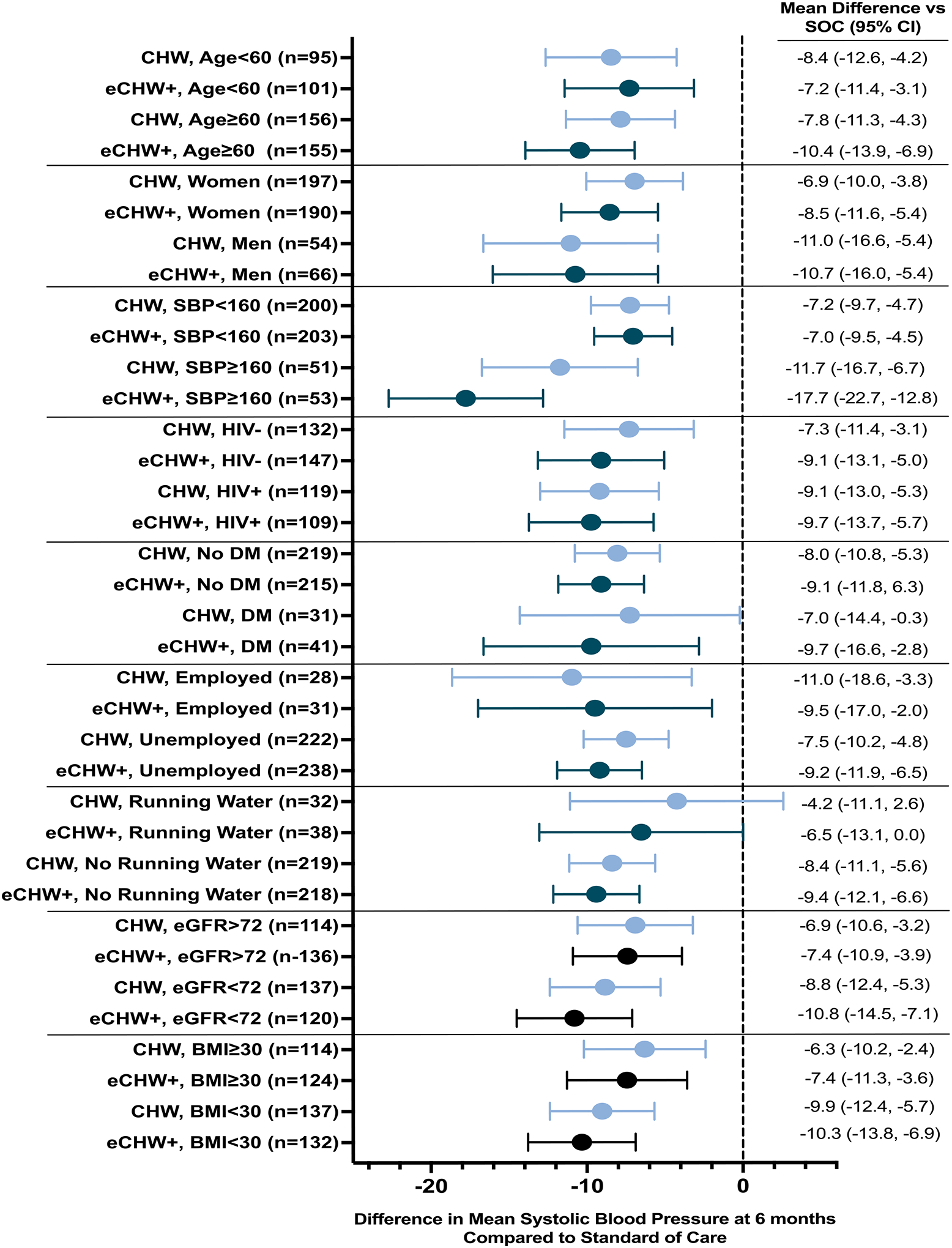
Estimated difference and 95% confidence interval in mean systolic blood pressure at 6 months between community health worker (CHW), enhanced community health worker (eCHW+) and standard of care (SOC) arms by sub-groups of interest DM: diabetes mellitus; eGFR: estimated glomerular filtration rate; BMI: body mass index

The magnitude of benefit in terms of mean systolic BP reduction at 6 months observed in the intervention groups compared to SOC was similar in most sub-groups. There appeared to be a greater effect in those with a systolic BP at enrollment ≥160mmHg versus those with a systolic BP at enrollment of 140–160 mmHg (Figure 2).

## Discussion

In a rural, low-resource region of South Africa, a home-based model of hypertension care, characterized by patient self-monitoring, CHW visitation, and remote, nurse-led decision making supported by a mobile health application, substantially reduced systolic BP and improved rates of hypertension control at 6 months. The 8–10mmHg reduction in mean systolic BP observed in the intervention arms has been associated with a 15–25% reduction in risk of heart attack, stroke, and heart failure.^17^ Improvements appeared to persist at 12 months and were evident irrespective of sociodemographic or clinical characteristics. These results in a historically disadvantaged community support home-based hypertension care in similar low-resource settings, and are consistent with community-based models for other chronic diseases^18^ and recommendations made by the South African Department of Health and World Health Organization.^2,8^

Our results differ from many prior studies of interventions using mobile health applications to enhance hypertension care in resource-limited settings. For example, a meta-analysis of 9 randomized controlled trials in resource-limited settings that compared in-person versus remote hypertension care estimated a mean difference in systolic BP of 1mmHg between arms.^19^ Interventions in that review included text messaging communication platforms, clinical decision support tools, and, in one study in China, provision of home-based BP devices to participants. Yet, in contrast to our study, no study in that review combined multiple strategies to address the multifactorial barriers to chronic disease care. A separate meta-analysis of non-pharmaceutical strategies to improve hypertension care observed substantial improvements with health systems approaches and more modest improvements with patient-focused approaches.^20^ Few of the data in that review were derived from low-resource settings. The COBRA BPS study evaluated a CHW-engaged model of care and documented a reduction in systolic BP of 5 mmHg compared to usual care.^21^ However, in that study, CHW involvement was limited to home-based BP measurement. Following the initial visit, participants traveled to clinic for ongoing care. An alternative approach using group-based care in Kenya resulted in reductions in systolic BP, but the reductions observed (3.3–3.9 mmHg) were not statistically different from those in the standard of care arm.^22^ Finally, a study among predominantly black men in the US compared clinic-based care with a pharmacist-led program in barbershops. They observed a 20mmHg reduction in mean systolic BP compared to standard care.^23^ Our study was similar in its focus on a population with historical inequities in healthcare access and use of an intervention targeting structural and socio-behavioral barriers. Our study differed by the use of a home-based care model and inclusion of both men and women. Our study was unique among this body of research as the only one evaluating a home-based intervention in which participants received BP monitoring and visitation by lay healthcare workers. As in our study, prior work has suggested that BP self-monitoring is more effective when paired with health system support.^24^

This study was strengthened by use of an effectiveness evaluation design to enhance generalizability for other remote and low-resource settings. For example, CHWs who participated in the program had the educational equivalent of high school diplomas and were recruited from local villages, as recommended by South African CHW recruitment policy. Clinical care for participants in the program was provided by nurses employed at public-sector primary clinics. Moreover, the study population was typical of those in many other resource-constrained settings: approximately one in three participants completed more than a primary education, only one in five had in-home access to water, and the mean transportation time to the nearest clinic was 45 minutes.

Our study is limited by its conduct in two clinics in one region of a single country. Studies in urban areas and settings without CHW programs will be needed to determine its effect in such locations. We also studied a population with established hypertension, most of whom were already on treatment. Although we found benefits of the intervention in both men and women, our study population was predominantly comprised of women. Interventions that better engage and retain men in hypertension care remain a high priority in South Africa.^25^ We also only studied one disease. Future work should consider the feasibility of expanding such programs to address multimorbidity. Adverse events were collected by recall at 6 monthly home visits, which may have led to under-reporting of minor adverse events and medication side-effects. Finally, the cost implications of the program to individuals and health systems are not yet known. In future work, we plan to estimate the cost-effectiveness of the intervention. This will include comparisons between health resource allocations and benefits between the CHW and eCHW+ arms, the latter of which entails more costly BP machines but fewer human resources due to automated transfer of BP data.

In summary, a home-based model of hypertension care reduced systolic BP and improved hypertension control in South Africa. Future work should consider such models in other resource-limited settings and expansion of the program to include care for people with multiple comorbidities. In the meantime, primary care programs with poor performance may consider similar remote models of care that address structural barriers to improve hypertension control.

## Supplementary Material

supp app

## Figures and Tables

**Table 1.** Demographic and clinical characteristics of study participants at enrollment

	SOC (n=259)	CHW (n=257)	eCHW+ (n=258)	Total (n=774)
**Age (years),** mean (SD)	62 (12)	63 (12)	62 (11)	62 (12)
**Female**	194 (74.9%)	202 (78.6%)	192 (74.4%)	588 (76.0%)
**Education level**				
None	103 (39.8%)	110 (43.0%)	90 (34.9%)	303 (39.2%)
Primary education	57 (22.0%)	61 (23.8%)	65 (25.2%)	183 (23.7%)
>Primary education	99 (38.2%)	85 (33.2%)	103 (39.9%)	287 (37.1%)
**Employment status**				
Not employed	227 (89.0%)	226 (89.0%)	225 (87.9%)	678 (88.6%)
**Asset Index Quintile**				
Most Deprived	64 (24.8%)	43 (17.1%)	55 (21.5%)	162 (21.2%)
Deprived	42 (16.3%)	54 (21.5%)	51 (19.9%)	147 (19.2%)
Moderate	58 (22.5%)	51 (20.3%)	42 (16.4%)	151 (19.7%)
Less Deprived	54 (20.9%)	46 (18.3%)	53 (20.7%)	153 (20.0%)
Least Deprived	40 (15.5%)	57 (22.7%)	55 (21.5%)	152 (19.9%)
**Running water in the household**				
No	218 (84.5%)	222 (86.7%)	220 (85.3%)	660 (85.5%)
**Travel time to clinic (minutes)**, mean (SD)	47 (43)	52 (187)	41 (33	47 (112)
Cost of travel to clinic (rand*), mean (SD)	26.21 (16.14)	30.58 (27.17)	29.11 (24.93)	28.68 (23.37)
**SBP at baseline (mmHg)**, mean (SD)	147.4 (16.4)	146.6 (18.0)	146.8 (17.2)	147.0 (17.2)
**SBP≥160 mmHg at enrolment**	50 (19.3%)	53 (20.6%)	53 (20.5%)	156 (20.2%)
**Taking antihypertensive therapy at enrollment**	251 (96.9%)	249 (96.9%)	251 (97.3%)	751 (97.0%)
**Body mass index (kg/m**^**2**^**)**, mean (SD)	29.3 (7.5)	29.8 (7.1)	30.0 (7.1)	29.7 (7.2)
**Glomerular filtration rate** (milliliters/minute)^±^, mean (SD)	75.5 (14.9)	74.5 (14.1)	78.2 (15.7)	76.1 (15.0)
**Diabetes**	32 (12.4%)	32 (12.5%)	41 (15.9%)	105 (13.6%)
**HIV status**				
Negative	131 (50.6%)	133 51.8%)	149 (57.8%)	413 (53.4%)
Positive	128 (49.4%)	123 (47.9%)	109 (42.2%)	360 (46.5%)
Unknown	0 (0.0%)	1 (0.4%)	0 (0.0%)	1 (0.1%)

SD: standard deviation; SOC: standard of care arm; CHW: community health worker arm; eCHW+: enhanced community health worker arm: SBP: systolic blood pressure

* At the time of the study $1 US was equivalent to approximately 18 South African rands

± Glomerular filtration rate was estimated using creatinine measurements on the day of enrollment with the CKD-EPI equation^11^

**Table 2.** Outcomes and adverse events by study arm

Outcome	SOC (n=259)	CHW (n=257)	eCHW+ (n=258)
Systolic blood pressure, mean (95% CI)			
Enrollment (n=774)	147.4 (145.4, 149.4)	146.6 (144.4, 148.8)	146.8 (144.7, 149.0)
6 months (n=762)	145.8 (143.4, 148.2)	137.5 (135.6, 139.4)	136.5 (134.8, 138.1)
Change from enrollment to 6 months	−1.9 (−4.2, 0.4)	−9.1 (−11.3, −6.8)	−10.5 (−12.8, −8.2)
Difference between arms at 6 months vs SOC	Reference	−7.9 (−10.5, −5.3)	−9.1 (−11.7, −6.4)
P-value vs SOC arm at 6 months	N/A	<0.001	<0.001
12 months (n=754)	144.8 (142.6, 147.1)	134.1 (132.6, 135.7)	134.0 (132.6, 135.4)
Change from enrollment to 12 months	−3.0 (−5.1, −0.9)	−12.4 (−14.7, −10.1)	−12.8 (−15.1, −10.5)
Difference between arms at 12 months vs SOC	Reference	−10.3 (−12.6, −8.0)	−10.5 (−12.8, −8.2)
Proportion with blood pressure control			
6 months (n=762), (%, 95%CI)	57.6% (51.5, 63.6)	76.9% (71.2, 81.7)	82.8% (77.7, 87.0)
Relative risk for hypertension control vs SOC	Reference	1.33 (1.18, 1.51)	1.44 (1.28, 1.62)
12 months (n=754), (%, 95%CI)	57.7% (51.5, 63.7)	82.8% (77.6, 87.0)	85.7% (80.7, 89.5)
Relative risk for hypertension control vs SOC	Reference	1.43 (1.27, 1.62)	1.48 (1.32, 1.67)
Safety events over observation period			
Total adverse events, n (%)	4 (1.6%)	7 (2.7%)	10 (3.9%)
Severe adverse events, n (%)	4 (1.6%)	7 (2.7%)	10 (3.9%)
Study-related adverse events, n (%)	0 (0%)	0 (0%)	0 (0%)
Deaths, n (%)	3 (1.2%)	1 (0.4%)	4 (1.6%)
Retention in care at 6 months, n (%)	76.4% (70.9, 81.2)	98.1% (95.4, 99.2)	99.6% (97.3, 99.9)
Retention in care at 12 months, n (%)	72.6% (66.8, 77.7)	97.3% (94.4, 98.7)	97.7% (94.9, 99.0)

SOC: standard of care arm; CHW: community health worker arm; eCHW+: enhanced community health worker arm: SBP: systolic blood pressure; CI: confidence interval
