# Supplementary material for: Home-Based Care for Hypertension in Rural South Africa
Source: N Engl J Med. Author manuscript; Available in PMC 2025 Sep 6. (PMC12412915; doi:10.1056/NEJMoa2509958)
Supplement: supp app [file NIHMS2107866-supplement-supp_app.pdf]

Supplementary Appendix 1

This appendix has been provided by the authors to give readers additional information about their work. It contains supplementary methods, tables, and figures.

Supplement to:

Home-based hypertension care in rural South Africa

Mark J. Siedner, Nombulelo Magula, Lusanda Mazibuko, Nsika Sithole, Alison Castle, Siyabonga Nxumalo, Thabang Manyaapelo, Shafika Abrahams-Gessel, Dickman Gareta, Joanna Orne-Gliemann, Kathy Baisley, Max Bachmann, Thomas A. Gaziano

Supplement Version Date: 5 August 2025

Supplementary Appendix 1 Table of Contents

Supplemental Methods 2
Author Contributions 5
Figure S1. Consort Diagram 6
Figure S2. Map of study catchment area 7
Figure S3. IMPACT-BP mobile health application interface 8
Table S1. Participant demographics 9
Table S2. Sensitivity analyses for systolic blood pressure outcome 10
Table S3. Sensitivity analyses for hypertension control outcome. 11
Table S4. Mean systolic and diastolic blood pressure 12
Table S5. Listing of all severe adverse events and deaths 13
Supplement References 14

Supplemental Methods

Recruitment and Randomization

We recruited participants from two public-sector primary health clinics in Northern KwaZulu-Natal, South Africa: the Nkundusi Clinic and Madwaleni Clinic. Individuals over 18 years of age presenting for primary care were screened with BP measurement. Those who had elevated BP were then further evaluated including a chart review to confirm a prior elevated room at least six months prior, point-of-care creatinine testing to determine renal function, and urine β-HCG testing for women under 55 years of age. Those who remained eligible and provided written informed consent were randomized 1:1:1 in blocks of 9 to one of the three trial arms using the randomization module in REDCap.[1,2] Randomization was stratified by clinic and current hypertension treatment at enrollment. The study statistician generated the randomization table and only the data manager had access to the locked randomization table. After randomization, both participants and clinic staff were aware of allocation.

Community Health Worker Recruitment and Selection

CHWs supported patient care in the two intervention arms. To ensure coverage of the clinic catchment areas, CHWs were recruited using South African Department of Health CHW guidelines. These guidelines require that CHWs are local community members and have completed a diploma (i.e. equivalent of a high school education). No prior healthcare training is required. The study recruited a total of 26 CHWs to provide service for a catchment area (Figure S2).

Mobile Health Application with Clinical Decision Support Tool

Both intervention arms were also supported by a mobile health application designed by the study team and developed by software engineers (iMarketing, Windhoek, Namibia). At enrollment, a nurse enters demographic information, drug allergies, medical co-morbidities (diabetes, heart disease, HIV), and hypertension treatment history in the application for each participant. The application has an interface for CHWs to enable entry of BP readings and their dates (Supplemental Figure S3A). For nurses, the application is pre-populated with a clinical decision support algorithm that replicates the South African Department of Health hypertension treatment guidelines, such that when a nurse logs into the application and reviews a patient, the mean BP from the prior two weeks of BP readings are displayed along with the current treatment regimen (Supplemental Figure S3B). This information is used to recommend a clinical action (Supplemental Figure S3C), as determined by South Africa Department of Health Guidelines. Recommendations can include no change in regimen, for example if the BP is controlled (i.e. <140mm Hg systolic and <90 Hg diastolic based on South African guidelines), an increase in the dose of a current medication, or the addition of a new medication. Treatment recommendations also take into account drug allergies and medical co-morbidities. Nurses have the option to over-ride the recommendation and select an alternate regimen and/or make a referral to a physician for further care. Once a treatment decision is made, enrolled nurses received a prompt in their version of the application to fill the ordered prescription. Once completed, study community health workers receive a prompt,

requesting them to pick up the prescription and deliver it to the relevant participant’s home (Supplemental Figure S3D).

Sample Size Considerations

Based on a recent population-based study of blood pressure in the area, we anticipated mean BP at baseline would be 150/95 mmHg, with a standard deviation (SD) of 19 mmHg.[3] With a target enrollment of 774 participants (258 per arm), we estimated >80% power to detect a 5 mmHg difference between arms in systolic BP at 6 months, assuming 20% loss to follow-up, a correlation between baseline and follow-up measurements of 0.5, and a two-sided alpha of 2.5% to account for multiple comparisons (two intervention arms vs SOC). This same sample size would also give us >80% power to detect an increase in the proportion of participants who achieve hypertension control at 6 months (defined by a systolic BP <140 mmHg and diastolic BP <90mm Hg) from 30% in the SOC arm to 45% in the interventions arms, or from 40% in the SOC to 56% in the intervention arms, also allowing for a two-sided alpha of 2.5% to account for multiple testing. Our estimation of approximately 30-40% control in the SOC arm was based on a large population-based study in the study catchment area which estimated approximately 40% prevalence of hypertension control.[3]

Study Outcomes

Our primary outcome of interest was systolic BP at 6 months. The secondary outcome of interest was the proportion of participants with hypertension control at 6 months, defined by a systolic BP <140mm Hg and a diastolic BP <90mm Hg. Safety outcomes included adverse events and retention in hypertension care, defined as an interaction with a healthcare worker (ie nurse, physician or CHW) for hypertension care within the past 3 months. Our pre-specified secondary outcomes were systolic BP at 12 months and proportion with hypertension control at 12 months.

Difference in systolic BP between treatment arms at 6 and 12 months

To compare the primary outcome by treatment arm, we fit linear regression models to estimate the mean difference between the SOC arm and each intervention arm (i.e. CHW vs SOC and eCHW+ vs. SOC) in systolic BP at 6 months. The response variable was systolic BP at 6 months and the model included treatment arm, systolic BP at enrollment, and the two stratification variables: clinic and use of hypertension medication at enrollment. We used the same approach for our secondary outcome of systolic BP at 12 months. We used an intention to treat approach for all analyses. In our primary analysis, those with missing data were censored.

Estimating Proportion with Blood Pressure Control at 6 and 12 months

To assess our secondary outcome of proportion with hypertension control by treatment arm, we fit logistic regression models and derived the relative risk (RR) using marginal standardization for the effect of each intervention on hypertension control, compared with SOC.[4] The model included terms for treatment arm and randomization stratification variables:

clinic and use of hypertension medication at enrollment. We then repeated this analysis with data from the 12-month timepoint.

Safety Measures

To compare safety outcomes, we summarized the number and severe of adverse events by study arm, including non-serious adverse events, serious adverse events, hospitalizations and deaths. We then calculated the proportion of participants retained in hypertension care, with 95% confidence intervals, at 6 and 12 months.

Sub-group Analyses

In exploratory analyses, we estimated the main treatment effect at 6 months by fitting an interaction term between treatment arm and pre-specified sub-groups of interest: sex, age <60 versus ≥60, systolic BP at enrollment <160mm Hg versus ≥160mm Hg, and HIV serostatus) to examine potential effect modification. In post-hoc analyses we also considered the effect sociodemographic variables, including employment status and whether participants had running water in their homes.

Sensitivity Analyses

We conducted three sets of pre-specified sensitivity analyses. First, we repeated the primary analysis but included people with missing data at 6 months, assuming no change in systolic BP from enrollment and lack of hypertension control, respectively. Second, we adjusted the analysis of mean systolic blood pressure at each timepoint for age, sex, body mass index (BMI) at enrollment and HIV serostatus at enrollment. Although it was specified in the analysis plan, smoking status was not included in these models because smoking history was not included in the study questionnaire. Active smoking prevalence was very low in this population in a recent population-based trial.[3] Finally, we also estimated the effect of each intervention versus SOC on mean systolic BP over 12 months using a linear mixed model. The response variable was systolic BP and included data at baseline, 6 and 12 months, using a constrained baseline approach (i.e. the treatment arm at baseline set to SOC for all participants).[5] The model included fixed effects for study arm, timepoint, a study arm-timepoint interaction term, randomization strata and baseline systolic blood pressure. Participant identification number was included a random effect to account for correlation of repeated measurements within individuals.   A similar approach was used to estimate the effect of each intervention on BP control over 12 months, using random effects logistic regression. The model included fixed effects for study arm, timepoint, a study arm-point interaction term, and randomization, and a random effect to account for correlation of repeated measurements within participants.

Author Contributions

Authored first draft of the manuscript: MJS

Contributed to study design: NM, LM, NS, AC, SN, TM, SAG, DG, JOG, KB, MB, TAG

Funding acquisition: MJS, NM, TAG

Data collection: NS, AC, TM

Data analysis: MJS, LM, KB

Provided critical revisions: NM, LM, NS, AC, SN, TM, SAG, DG, JOG, KB, MB, TAG

Figure S1. Consort Diagram

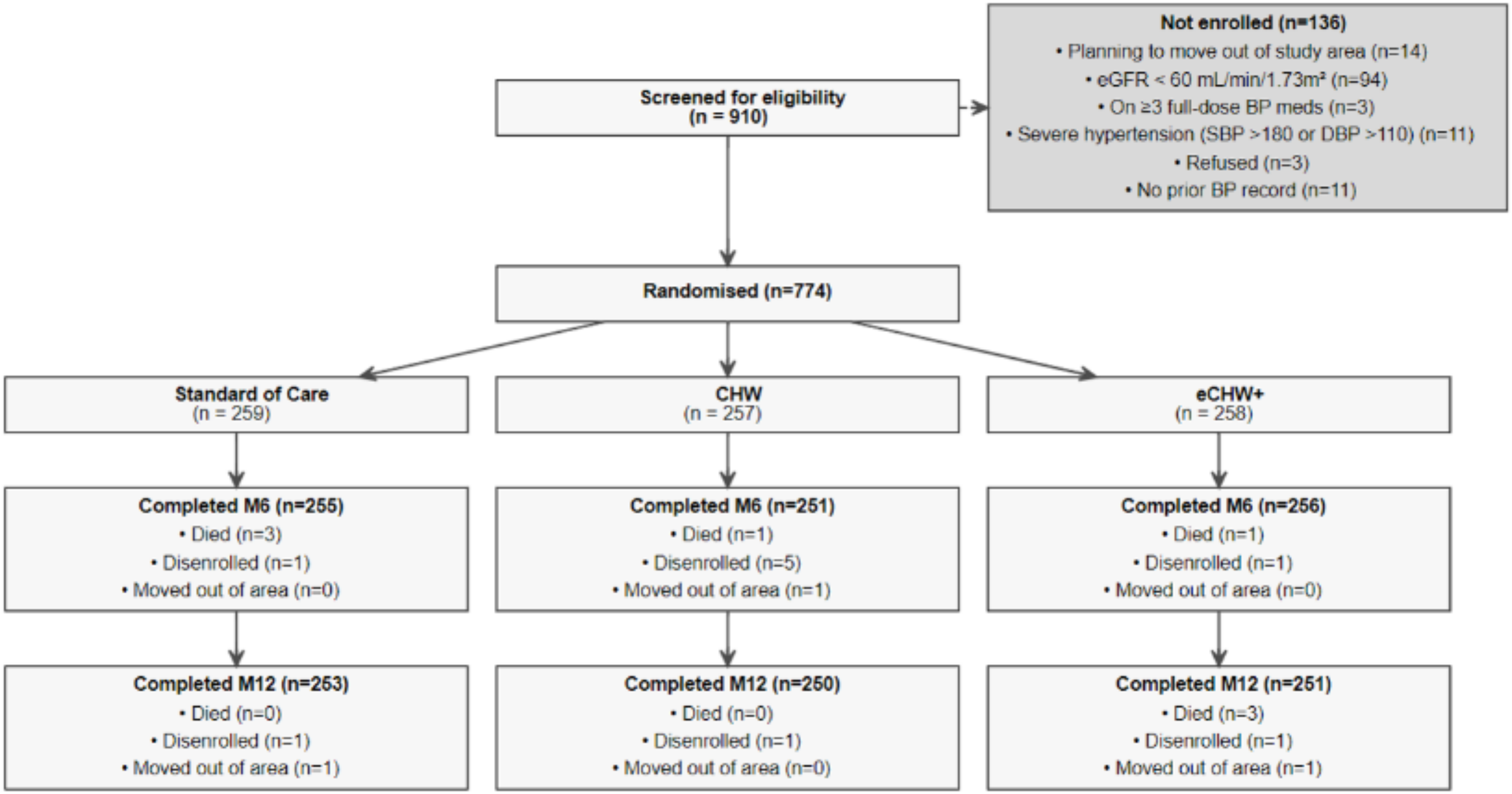

Figure S2. Map of study catchment area denoting location of clinics and residences of community health workers

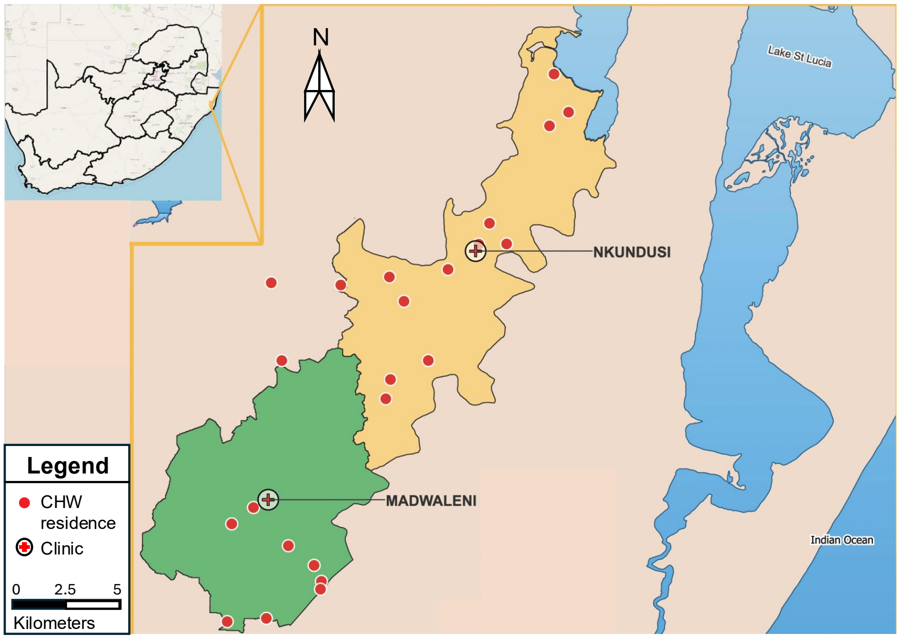

Figure S3. IMPACT-BP mobile health application interface. Screenshots show examples of the blood pressure recording page (S3A), the nursing blood pressure review page (S3B), the nursing clinical decision support tool (S3C) and the pharmacy prescription confirmation page to alert community health workers that medicines are ready for pick-up (S3D).

S3A. Blood pressure entry interface

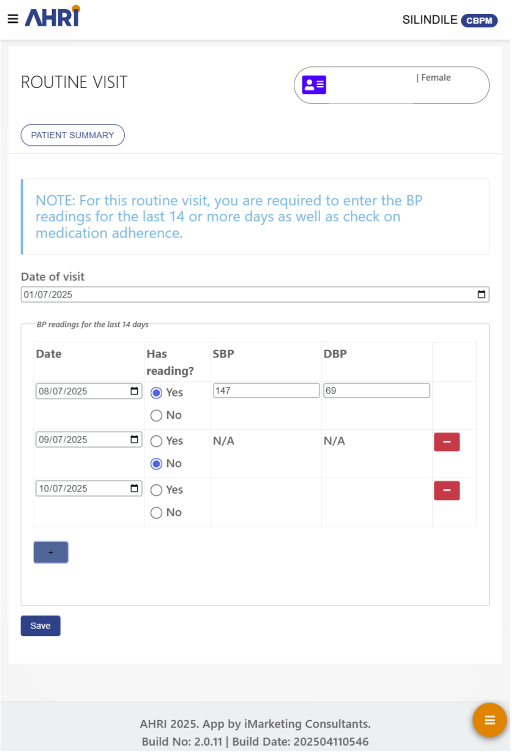

S3B. Blood pressure history interface

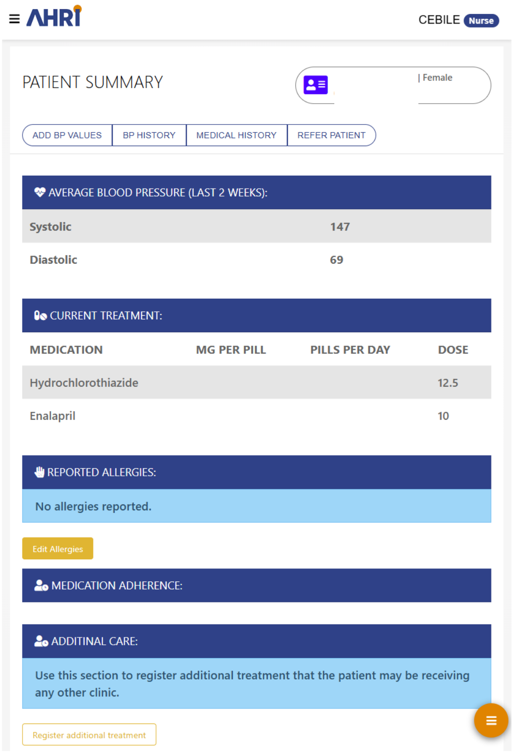

S3C. Clinical decision support interface

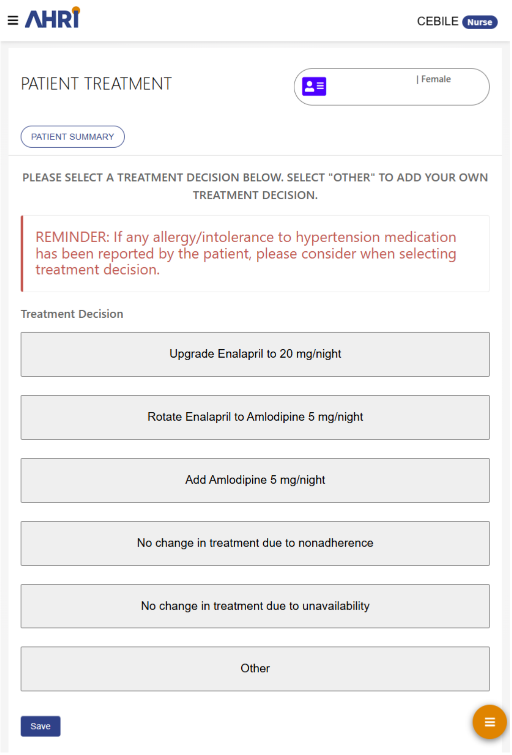

S3D. Medication confirmation interface

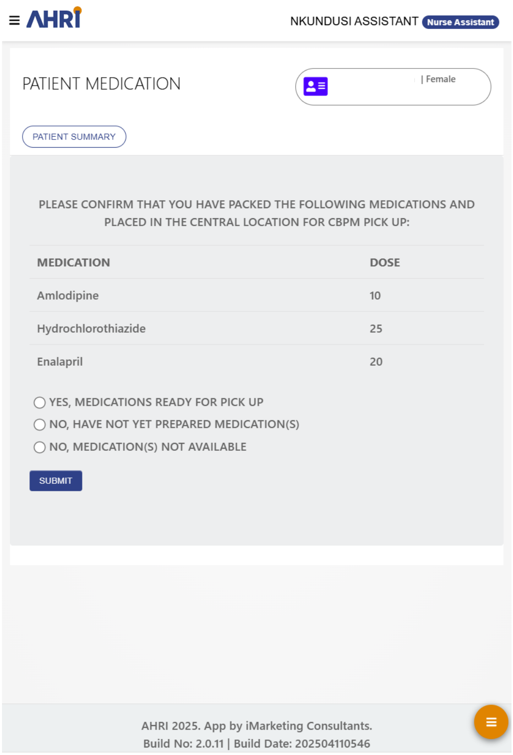

Table S1. Participant demographics and demographics of people living with hypertension, derived from a population-based study in the same catchment area.3 Demographic data are collected for all individuals in the study catchment area through a demographic health and surveillance program.[6]

	IMPACT-BP Study Participants (n=774)	People with Uncontrolled Hypertension in the Study Catchment Area (n=2,680)
Age (n, %)		
0-17	0	50 (1.9%)
18-29	2 (0.3%)	181 (6.8%)
30-39	25 (3.2%)	257 (9.6%)
40-49	77 (10.0%)	335 (12.5%)
50-59	209 (27.0%)	572 (21.3%)
60-69	267 (34.5%)	656 (24.5%)
70-79	137 (17.7%)	406 (15.1%)
80+	57 (7.4%)	223 (8.3%)
Sex (n, %)		
Male	186 (24.0%)	751 (28.0%)
Female	588 (76.0%)	1,929 (72.0%)
Race/Ethnicity (n, %)		
Asian, non-Hispanic	0 (0%)	0 (0%)
Black	774 (100%)	2,680 (100%)
White, non-Hispanic	0 (0%)	0 (0%)
Other	0 (0%)	0 (0%)

Table S2. Sensitivity analyses for systolic blood pressure outcome. Linear regression models estimating the difference in mean systolic BP between arms in primary analytic model (Model 1), a model in which participants with missing BP data at 6 months have baseline reading carried forward (Model 2), a model with additional adjustment for pre-specified factors (Model 3), and a random effects model with data from both 6 and 12 month timepoints (Model 4).

	**Model 1: Primary Model**	**Model 2: Last Blood Pressure Carried Forward**	**Model 3: Fully Adjusted Model**	**Model 4: Random Effects Model**
**Characteristic**	**Linear Coefficient (95% CI)**	**Linear Coefficient (95% CI)**	**Linear Coefficient (95% CI)**	**Linear Coefficient (95% CI)**
**Systolic BP at 6 months**				
Standard of care	Reference	Reference	Reference	Reference
CHW	–7.9 (–10.5, –5.3)	–7.5 (–10.1, –4.9)	–8.0 (–10.7, –5.4)	-7.9(-10.4, -5.3)
eCHW+	–9.1 (–11.7, –6.4)	–8.9 (–11.5, –6.3)	–9.1 (–11.7, –6.4)	-9.0 (-11.6, -6.5)
**Systolic BP at 12 months**				
Standard of care	N/A	N/A	N/A	Reference
CHW	N/A	N/A	N/A	-10.2 (-12.8, -7.7)
eCHW+	N/A	N/A	N/A	-10.5 (-13.1, -7.9)

BP: blood pressure; CI: confidence interval; CHW: community health worker arm ; eCHW+: enhanced community health work arm

Table S3. Sensitivity analyses for hypertension control outcome. We used logistic regression models with marginalized standardization to estimate the relative risk of hypertension control by study arm in the primary analytic model (Model 1), a model in which participants with missing data at 6 months are presumed not to have hypertension control (Model 2), a model with additional adjustment for pre-specified factors (Model 3)*, and a random effects model with data from both 6 and 12 month timepoints (Model 4).

Characteristic	Model 1: Primary Model<br>Relative Risk (95% CI)	Model 2: Last Blood Pressure Carried Forward<br>Odds Ratio (95% CI)	Model 3: Fully Adjusted Model<br>Odds Ratio (95% CI)	Model 4: Random Effects Model<br>Odds Ratio (95% CI)
**Systolic BP at 6 months**				
Standard of care	Reference	Reference	Reference	Reference
CHW (CBPM)	1.33 (1.18, 1.51)	1.32 (1.16, 1.50)	1.33 (1.17, 1.51)	1.32 (1.16, 1.49)
eCHW+ (eCBPM+)	1.44 (1.28, 1.62)	1.45 (1.28, 1.63)	1.43 (1.27, 1.61)	1.42 (1.27, 1.60)
**Systolic BP at 12 months**				
Standard of care	N/A	N/A	N/A	Reference
CHW (CBPM)	N/A	N/A	N/A	1.42 (1.26, 1.59)
eCHW+ (eCBPM+)	N/A	N/A	N/A	1.47 (1.31, 1.65)

BP: blood pressure; CI: confidence interval; CHW: community health worker arm ; eCHW+: enhanced community health work arm
*Model 3 was additionally adjusted for age, sex, body mass index at enrollment and HIV serostatus at enrollment.

Table S4. Mean systolic and diastolic blood pressure by study arm and timepoint.

	Standard of Care	CHW	eCHW+
Enrollment			
Systolic BP (mean, SD)	147.4 (16.3)	146.6 (18.0)	146.8 (17.2)
Diastolic BP (mean, SD)	90.8 (10.9)	90.9 (10.5)	91.1 (10.0)
6 months			
Systolic BP (mean, SD)	145.8 (19.3)	137.5 (15.2)	136.5 (13.6)
Diastolic BP (mean, SD)	90.2 (10.9)	86.7 (8.9)	86.1 (8.5)
12 months			
Systolic BP (mean, SD)	144.8 (18.2)	134.1 (12.3)	134.0 (11.3)
Diastolic BP (mean, SD)	90.2 (10.6)	84.9 (8.1)	84.8 (8.5)

BP: blood pressure; SD: standard deviation; CHW: community health worker arm; eCHW+: enhanced community health work arm

Table S5. Listing of all severe adverse events and deaths by study arm during the observation period

Study Arm	Event Type	Days from Enrollment	Description	Related to Study Procedures as Adjudicated by Investigators
Standard of Care	Death	62	Found dead at home by a family member. No further details of the death were available.	Not related
Standard of Care	Hospitalization	123	Hospitalized for high blood pressure and dyspnea.	Not related
Standard of Care	Death	100	Died at home after weeks of worsening shortness of breath that did not improve with treatment provided at the clinic.	Not related
Standard of Care	Death	302	Died at home after sudden onset of vomiting and abdominal distension.	Not related
CHW	Hospitalization	3	Hospitalized for elevated blood sugar.	Not related
CHW	Hospitalization	166	Hospitalized for elective eye surgery.	Not related
CHW	Hospitalization	345	Hospitalized for initiation of anticoagulants	Not related
CHW	Hospitalization	256	Hospitalized for excessive vaginal bleeding.	Not related
CHW	Hospitalization	234	Hospitalized days for complications of severe hip pain.	Not related
CHW	Death	207	Hospitalized and ultimately died with diagnosis of renal cancer and renal failure.	Not related
CHW	Hospitalization	59	Hospitalized after a fall for arm fracture	Not related
eCHW+	Hospitalization	326	Hospitalized for arm fracture	Not related
eCHW+	Hospitalization	145	Hospitalized for foot injury	Not related
eCHW+	Death	228	Death from traumatic injury	Not related
eCHW+	Hospitalization	129	Hospitalized for palpitations	Not related
eCHW+	Death	34	Died after hospitalization from complications of cholelithiasis and heart failure	Not related
eCHW+	Hospitalization/Death	165/256	Hospitalized after syncopal episode. Separate episode of admission for fatigue during which they died in hospital of unknown cause	Not related
eCHW+	Hospitalization	41	Hospitalized for urinary retention	Not related
eCHW+	Hospitalization	278	Hospitalized for vaginal bleeding and cervical cancer	Not related
eCHW+	Death	268	Died at home after sudden onset of dyspnea and chest pain	Not related

CHW: community health worker arm ; eCHW+: enhanced community health work arm

Supplement References

1. Sithole N, Castle A, Nxumalo S, et al. Protocol: Implementation Evaluation of a Combination Intervention for Sustainable Blood Pressure Control in Rural KwaZulu-Natal, South Africa (IMPACT BP): A three-arm, unblinded, parallel group individually randomized clinical trial. Contemp Clin Trials 2023;131:107258.

2. Harris PA, Taylor R, Minor BL, et al. The REDCap consortium: Building an international community of software platform partners. Journal of Biomedical Informatics 2019;95:103208.

3. Wong EB, Olivier S, Gunda R, et al. Convergence of infectious and non-communicable disease epidemics in rural South Africa: a cross-sectional, population-based multimorbidity study. Lancet Glob Health 2021;9(7):e967–76.

4. Muller CJ, MacLehose RF. Estimating predicted probabilities from logistic regression: different methods correspond to different target populations. International Journal of Epidemiology 2014;43(3):962–70.

5. Coffman CJ, Edelman D, Woolson RF. To condition or not condition? Analysing ‘change’ in longitudinal randomised controlled trials. BMJ Open 2016;6(12):e013096.

6. Gareta D, Baisley K, Mngomezulu T, et al. Cohort Profile Update: Africa Centre Demographic Information System (ACDIS) and population-based HIV survey. Int J Epidemiol 2021;50(1):33–4.
